# Building an Endovascular Skeleton: Prophylactic Stenting in a Hybrid Approach to a Thymic Carcinoma with Supra-aortic Trunk Encasement

**DOI:** 10.1016/j.ejvsvf.2026.08.002

**Published:** 2026-08-22

**Authors:** Giulia Landini, Marco Campolmi, Alessandro Gonfiotti, Sara Speziali

**Affiliations:** a Section of Vascular Surgery, Department of Excellence of Experimental and Clinical Medicine, University of Florence, Florence, Italy; b Section of Thoracic Surgery, Department of Excellence of Experimental and Clinical Medicine, University of Florence, Largo Alessandro Brambilla, 3, 50134, Florence, Italy

**Keywords:** Carotid–subclavian bypass, Hybrid surgery, Intra-arterial stenting, Oncology, Supra-aortic trunks, Thymic carcinoma

## Abstract

**Introduction:**

Locally advanced thymic carcinomas with supra-aortic vessel involvement pose significant surgical challenges and are historically often deemed unresectable. Neoadjuvant radiotherapy further complicates surgical resection by inducing severe tissue fibrosis. The integration of endovascular techniques with open surgery can expand the boundaries of oncological radicality; however, literature on such hybrid approaches for mediastinal tumours remains limited.

**Report:**

A 45 year old man presented with a 6 × 9 cm locally advanced thymic squamous cell carcinoma causing tracheal compression. The mass entirely encased the left common carotid artery (LCCA) and partially encased the innominate and left subclavian arteries. Following a partial response to neoadjuvant chemoradiotherapy, a staged multidisciplinary surgical approach was planned. To mitigate the risk of catastrophic vascular injury during dissection of irradiated tissues, prophylactic stent grafts were first deployed in the innominate and left subclavian arteries via right axillary access under local anaesthesia. Radical tumour resection was performed via a combined median sternotomy and cervical approach three days later. The pre-placed stents acted as a rigid “endovascular skeleton”, allowing safe and aggressive sub-adventitial tumour dissection. The LCCA was resected *en bloc* with the tumour, and vascular continuity was restored using a left subclavian to carotid prosthetic bypass. The patient recovered well and was discharged on post-operative day 19. At the three month follow up, he was asymptomatic, with imaging confirming the patency of both the stents and the bypass.

**Conclusion:**

Prophylactic endovascular stenting of partially encased supra-aortic vessels offers crucial structural and haemostatic protection, facilitating safe and aggressive oncological resection in hostile, irradiated fields. This tailored, hybrid multidisciplinary approach provides a viable pathway to achieving true oncological radicality (R0) in highly complex mediastinal tumours previously considered inoperable.

## Highlights

• A rare case of thymic carcinoma with complete supra-aortic trunk encasement.
• Novel staged hybrid strategy combining endovascular and open surgical repair.
• Prophylactic endovascular stenting enabled safe radical oncological resection.
• Multidisciplinary teamwork is key to complex vascular involved malignancies.
• This approach expands surgical options for locally advanced mediastinal tumours.

## Introduction

Thymomas and thymic carcinomas are rare mediastinal tumours, with an incidence of 1.7 per million per year in Europe.^1^ Thymomas are usually slow growing with limited invasiveness, while thymic carcinomas are more aggressive, invading adjacent structures in up to 80% of cases. Complete surgical resection remains the mainstay of treatment at all stages and decisions about surgical treatment should be mainly based on two variables: the risk of incomplete resection and the patient's surgical risk.^1^, ^2^, ^3^ The adoption of modern hybrid strategies, integrating endovascular bridging techniques with open surgical reconstruction, has enabled true oncological radicality (R0) even in highly complex cases^3^, ^4^, ^5^ historically considered unresectable due to major vascular involvement.

**Figure 1:**
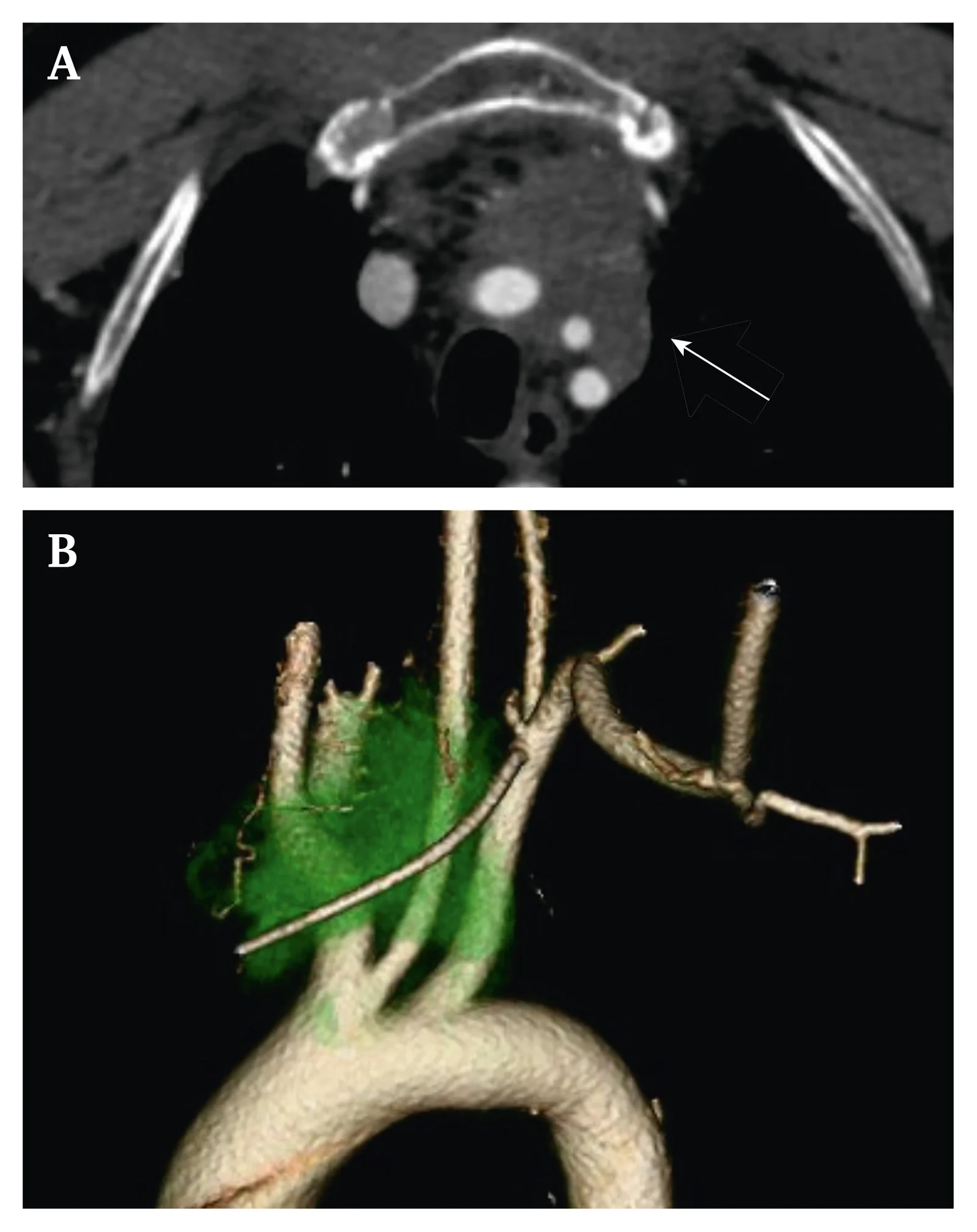
Pre-operative images. (A) Cross sectional view showing circumferential involvement of the left common carotid artery and only partial involvement of the brachiocephalic artery and left subclavian artery (white arrow); (B) Pre-operative three dimensional reconstruction. The thymic carcinoma is shown in green, circumferentially encasing the supra-aortic vessels.

**Figure 2:**
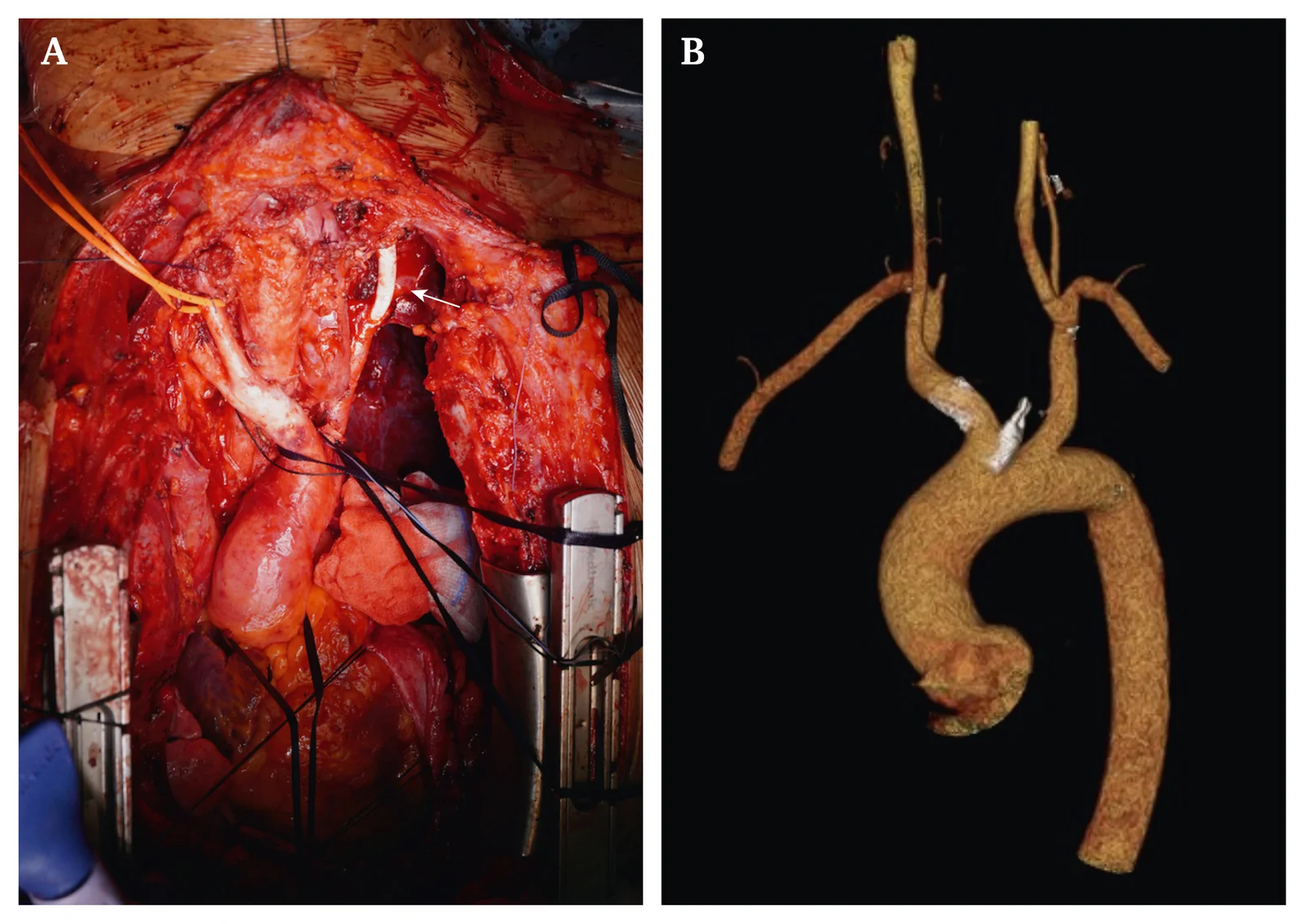
Final result after thymoma resection and left subclavian carotid bypass. (A) Intra-operative view showing the left subclavian artery to left common carotid artery prosthetic bypass (white arrow). (B) Three dimensional reconstruction from follow up computed tomography angiography.

**Figure 3:**
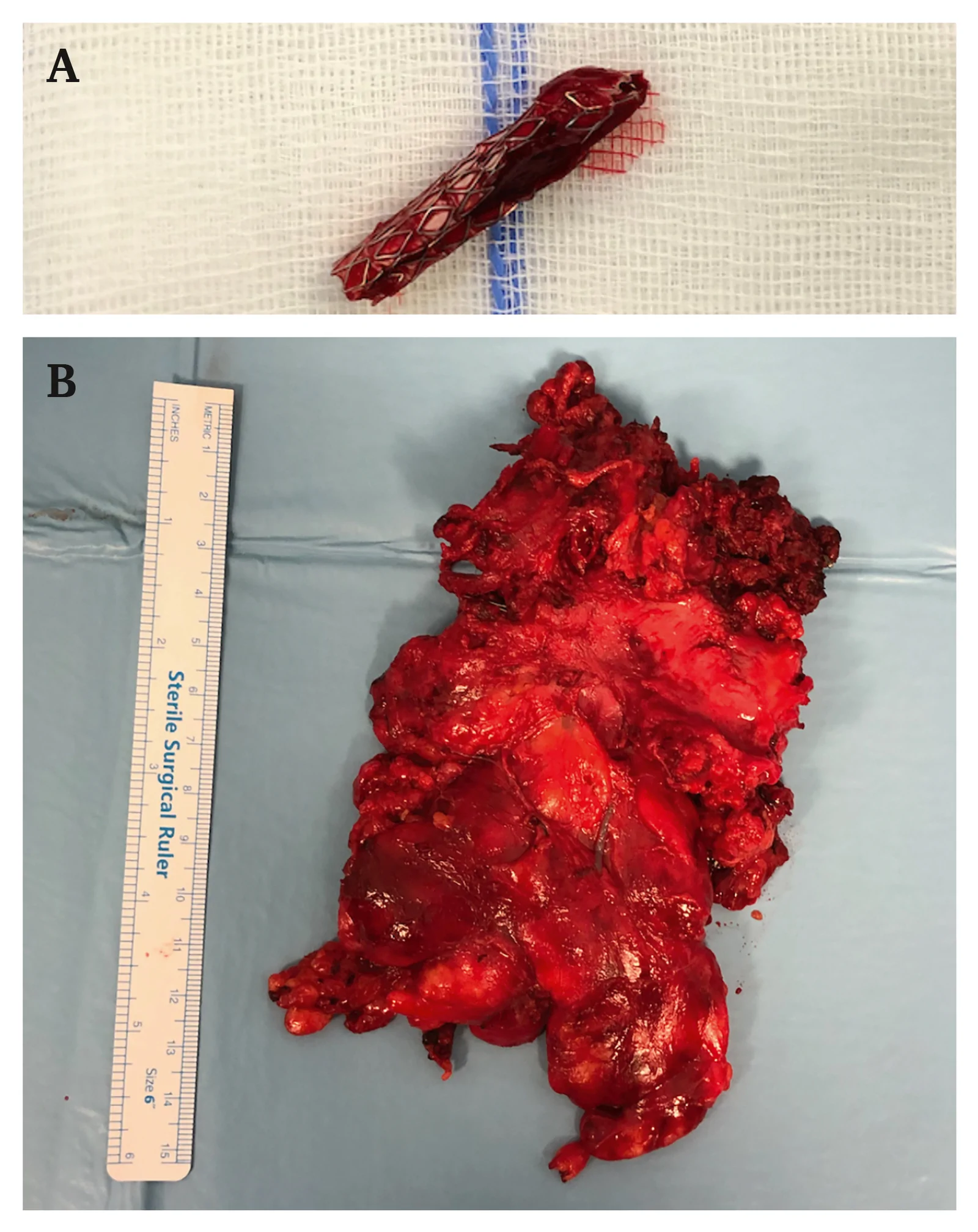
Post-operative images showing (A) Prophylactic carotid stent, which was previously deployed and subsequently removed from the left common carotid artery; (B) Resected thymic tumour mass.

Due to frequent involvement of mediastinal great vessels, a multidisciplinary approach is often necessary. For advanced or initially unresectable cases, multimodal therapy including chemotherapy, surgery, and sometimes radiotherapy is recommended.^6^, ^7^, ^8^, ^9^

This case report presents a rare case of locally advanced thymic squamous cell carcinoma with vascular involvement, successfully treated through a multidisciplinary, staged surgical approach.

### Report

A 45 year old man with no previous medical or surgical history presented with new onset neck pain and dysphonia (with subsequent aphonia). Neck ultrasound revealed a mass in the left upper mediastinum. Computed tomography angiography (CTA) showed a 6 × 9 cm multilobulated mass located below the thyroid gland, displacing the trachea to the right. The lesion involved the left brachiocephalic vein, and the left common carotid artery entirely, and partially encased the innominate and left subclavian arteries (Fig. 1). Positron emission tomography with fluorodeoxyglucose revealed mild hypermetabolism of the lesion.

Following thoracic surgical consultation and a biopsy via a small neck incision, histological analysis confirmed a non-keratinising squamous cell carcinoma. The immunohistochemical profile (AE1/AE3+, p63+, CD117+, CD5+, GLUT-1+, NUT-, CD20-, CD3-, CD30-) was strongly suggestive of a thymic origin (type C according to the World Health Organization system, stage III according to Masaoka-Koga staging).^9^

The case was discussed in a multidisciplinary team (MDT), which recommended neoadjuvant chemoradiotherapy to reduce tumour burden. After eight weeks, imaging showed partial tumour shrinkage, but persistent involvement of the supra-aortic vessels. Radical surgical resection was therefore indicated. Given the complexity of airway management in the supine position due to tracheal compression, the case involved detailed pre-operative planning by thoracic, vascular, and cardiac surgeons, and anaesthetists.

During the MDT evaluation, imaging confirmed that the left common carotid artery (LCCA) was 360° encased by the tumour, necessitating *en bloc* resection and reconstruction. In contrast, the innominate artery (IA) and left subclavian artery (LSA) were only partially encased. However, because the patient had undergone pre-operative neoadjuvant radiotherapy, the MDT anticipated severe tissue fibrosis and obliteration of safe surgical dissection planes. Pre-emptive stenting of these vessels was deliberately chosen to create a rigid, protective endovascular skeleton. This allowed the surgical team to perform an aggressive, radical dissection in irradiated, compromised tissues without the risk of inadvertent catastrophic injury to the partially encased major vessels.

The first stage consisted of prophylactic endovascular stenting of the supra-aortic vessels using three Viabahn VBX Balloon Expandable Endoprostheses (W. L. Gore & Associates, Flagstaff, AZ, USA), via a single right percutaneous axillary artery access with an 8 F introducer under local anaesthesia. Specifically, an 8 × 59 mm stent graft was deployed in the LCCA, an 11 × 39 mm stent graft in the LSA, and a 10 × 29 mm stent in the IA; no stent grafts were deployed across the ostia of aortic arch vessels. Right axillary access was chosen because the mediastinal bulk of the tumour caused severe dyspnoea, preventing the patient from lying fully supine unless intubated. Therefore, the procedure was carried out in a semi-seated position under local anaesthesia, reserving endotracheal intubation and general anaesthesia for the second, more invasive surgical phase. During this procedure, cerebral protection systems were not used, as the pathology was non-atherosclerotic and previous imaging studies had shown no critical lesions or stenosis at the level of the aortic arch and the supra-aortic vessels.

Following the initial endovascular step, maintaining stent patency while preparing for imminent major open surgery presented a distinct challenge. An aggressive but rapidly reversible antithrombotic bridging strategy was initiated, consisting of intravenous cangrelor, oral aspirin, and low molecular weight heparin. The use of cangrelor provided necessary protection against acute in stent thrombosis while allowing for rapid reversal and normalisation of platelet function immediately before the radical resection scheduled three days later.

Three days later, the patient underwent the second stage of surgery: radical tumour resection via median sternotomy combined with a cervical (Kocher) approach. The thoracic surgery team performed the resection with the vascular surgeons. The mass was carefully dissected, and due to direct infiltration and absence of electrical responsiveness, the left phrenic and left recurrent laryngeal nerves were transected. The left brachiocephalic vein was also ligated and divided due to tumour invasion.

Given the circumferential involvement of the LCCA, its proximal portion and the previously placed stent were resected *en bloc* with the tumour mass, as planned by the MDT. Since there was a significant distance between the two vessels after the resection, direct re-implantation of the carotid was ruled out. Therefore, intra-operatively, left subclavian to carotid bypass using a 6 mm Propaten graft (W. L. Gore & Associates) was selected. The subclavian to graft anastomosis was performed in an end to side configuration, distal to the left vertebral artery and the stented zone. The graft to carotid anastomosis was performed in an end to end fashion on the common carotid artery stump (Fig. 2A).

Meticulous dissection of the IA and the LSA allowed *en bloc* resection of the tumour. A cardiac surgery and perfusion team were kept on standby throughout the procedure to promptly manage any unexpected vascular complications, such as the need for cardiopulmonary bypass or complex heart and aortic reconstruction.

The post-operative course was uneventful. The patient remained in the intensive care unit for four days before being transferred to the ward. CTA on post-operative day three showed a patent carotid subclavian bypass and patent endovascular stents. Subtotal thrombosis of the left subclavian vein proximal to brachiocephalic venous clamping and focal thrombosis of the left internal jugular vein were noted. Due to phrenic nerve resection, the left hemidiaphragm was elevated, although without clinically significant respiratory compromise.

The patient was discharged on post-operative day 19 with dual antiplatelet therapy and low molecular weight heparin.

Final pathological analysis of the excised tumour (Fig. 3A) revealed a non-keratinising squamous cell carcinoma with a p40+, CD5+, and CD117+ immunophenotype; isolated lymph nodes showed no evidence of tumour involvement.

At the three month follow up, significant improvements in dysphonia and respiratory symptoms were noted. Follow up CTA at three months (Fig. 2B) demonstrated resolution of the previously observed left subclavian and jugular vein thromboses, with maintained patency of the carotid–subclavian bypass and endovascular stents.

## Discussion

Vascular invasion in thymic carcinoma, particularly involving the aorta and its major branches, represents a significant surgical challenge. Historically managed with cardiopulmonary bypass and extensive resection,^4^^,^^5^^,^^9^ such cases now benefit from hybrid approaches combining open surgery with pre-operative endovascular stent placement,^10^^,^^11^ which reduces the need for extensive vascular dissection even when a clear surgical plane is absent. Open surgery remains the gold standard for large, locally advanced tumours. Complete resection is the cornerstone of curative treatment, and multimodal neoadjuvant strategies are increasingly being employed to downstage tumours and improve resectability, although radiotherapy may increase tissue fibrosis and complicate subsequent dissection.

In this case, despite a partial response to neoadjuvant therapy, persistent tumour encasement of the supra-aortic trunks necessitated an individualised surgical plan. While once considered a contraindication to surgery, vascular involvement no longer precludes resection thanks to hybrid surgical techniques, underlining the value of vascular surgical planning within the MDT. For this patient, the vascular component of the decision making was driven by the anticipated effects of neoadjuvant radiotherapy. Irradiated tissues typically exhibit dense fibrosis, which obliterates standard peri-adventitial dissection planes and makes the separation of tumour from partially encased arteries highly hazardous.

In this context, prophylactic stenting of the partially involved LSA and IA was performed to provide critical protection through two distinct mechanisms. Firstly, the deployment of rigid stent grafts created a palpable endovascular skeleton. In a frozen, fibrotic mediastinum where visual landmarks are lost, this rigid core provides a constant tactile guide for the surgeon, minimising the risk of inadvertent iatrogenic transections. Secondly, the stent graft provides an additional layer of structural and haemostatic protection, allowing the surgeon to safely navigate the adventitial plane and aggressively shave tumour directly off the vessel. Such an approach enables the pursuit of true oncological radicality (R0) even in fibrotic and hostile surgical fields. Should the arterial wall become compromised or inadvertently breached during aggressive dissection, the internal stent graft ensures immediate haemostasis and vessel integrity, preventing rupture and the need for emergency vascular clamping.

Multidisciplinary collaboration among thoracic, vascular, and cardiac surgeons, and anaesthetists was essential. Careful anaesthetic planning was critical given the risk of airway compromise in the supine position; the initial endovascular procedure was therefore performed under local anaesthesia in a semi-upright position. Notably, the carotid subclavian bypass avoided the need for aortic clamping, reducing intra-operative risk.

This case supports the feasibility of hybrid strategies in advanced thymic malignancies, demonstrating how tailored vascular preconditioning can expand surgical indications in patients previously deemed unresectable. Prophylactic stenting, combined with radical resection and vascular reconstruction, represents a promising paradigm that merits further investigation. Long term follow up remains necessary to evaluate oncological control and durability of vascular reconstructions.

## Funding

This research did not receive any specific grant from funding agencies in the public, commercial, or not-for-profit sectors.

## Conflict of interest statement

The authors declare no conflict of interest.
